# Predictors of the length of stay of psychiatric inpatients: protocol for a systematic review and meta-analysis

**DOI:** 10.1186/s13643-021-01616-6

**Published:** 2021-03-02

**Authors:** Farid Carranza Navarro, Neri Alejandro Álvarez Villalobos, Andrea Muriel Contreras Muñoz, Andrea Fernanda Guerrero Medrano, Natalia Sofía Tamayo Rodríguez, Erasmo Saucedo Uribe

**Affiliations:** 1 Department of Psychiatry, Hospital Universitario “Dr. José E. González”, Av. Francisco I. Madero & Av. Gonzalitos, Mitras Centro, 64460 Monterrey, Mexico; 2 Plataforma INVEST KER Medicina UANL KER Unit Mayo Clinic (KER Unit Mexico), Universidad Autónoma de Nuevo León, Av. Francisco I. Madero & Av. Gonzalitos, Mitras Centro, Monterrey, Mexico

## Abstract

**Background:**

Length of stay (LOS) for inpatient psychiatric services is an important factor with serious drawbacks when it is extended more than needed. Impacts on economy, social functioning, and stigma can hamper improvement and affect the patients’ experiences on future mental healthcare. Predictions of which patients have a higher chance for prolonged LOS have been extensively researched. Previous systematic reviews found consistent predictors of both longer and shorter LOS. However, they do not provide an estimate from the pooled effect sizes. Furthermore, to our knowledge, there is no meta-analysis on the influence of these factors. The primary objective of this study will be to provide point estimates on the effect sizes of all studied predictors of the LOS of psychiatric inpatients.

**Methods:**

We will conduct a systematic search in PubMed, MEDLINE, EMBASE, and PsycINFO for observational studies evaluating the effect size of independent factors on the length of stay of psychiatric inpatients. Prospective and retrospective cohorts that assess the influence of predictors through the reporting of standardized regression coefficients will be included. We will provide a qualitative synthesis of the findings from each study and perform a meta-analysis from pooled regression coefficients that were adjusted for other variables or confounders in order to obtain a point estimate and confidence interval for all factors extracted from the included studies.

**Discussion:**

The results from this study may provide more accurate predictions for mental health institutions, psychiatrists, mental health service providers, patients, and families on the prognosis regarding the length of stay for needed inpatient care. This information may be used to anticipate individuals with a higher chance for prolonged hospitalization to plan the necessary interventions for these specific situations. Considering both the benefits and disadvantages of longer and shorter stays, the pooled estimates for independent factors may be used by mental healthcare providers and patients for informed decision-making. The results from this study will also update results presented in previous studies and identify the strengths and limitations from the current available evidence.

**Systematic review registration:**

PROSPERO ID CRD42020172840

**Supplementary Information:**

The online version contains supplementary material available at 10.1186/s13643-021-01616-6.

## Background

Hospitalization remains a vital intervention for patients with severe mental disorders [1]. In spite of its benefits, the experience of staying in a psychiatric hospital frequently is associated with low satisfaction [2], which may influence the patients’ attitude towards further mental healthcare [3]. One of the core determinants of this experience is the length of stay (LOS), defined as the number of days between discharge and admission.

Trends of the LOS for psychiatric inpatient care vary according to the geographic region. A recent prospective cohort of high-income European nations reported that the average LOS for hospitalized psychiatric patients was 39.4 days, ranging from 17.9 mean days in Italy to 55.1 mean days in Belgium [4]. In the USA, the maximum reported mean LOS in a systematic review of 30 studies was 24.9 days [5], 2 weeks shorter compared to other high-income countries in Europe. Comparatively, a prospective study of 385 psychiatric inpatients in Brazil reported a median LOS of 25 days [6]. A cross-sectional study in Nepal determined that the average LOS was 19.4 days, 20 days less compared to the mean in Europe [7]. While in most countries the duration of the hospital stay is less than 40 days, there is a clear variability between low-, middle-, and high-income nations, in accordance with the availability of resources.

The effects of the LOS on multiple outcomes in psychiatric inpatients have been extensively studied. As more studies continue to contribute to the large body of evidence, it has become clearer that longer stays are usually associated with more negative outcomes. These include deterring social and community-related skills [8], increased suicidal ideation related to stigma [9], social functioning [10], post-traumatic stress [11], and productivity and finances [12]. Although an ideal LOS remains uncertain, current international recommendations advocate for an early discharge as soon as stabilization is successful, in order to continue their management in a less restrictive environment [13]. On the contrary, concerns on prioritizing shorter admissions include an increase in medical negligence and favoring a “revolving-door” pattern. A Cochrane review from 2014 concluded that patient outcomes and rates of readmissions were similar between short and long LOS, albeit the quality of the evidence was low to very low [14]. This does suggest, however, that planning shorter admissions does not increase readmissions or the risk of discharging patients without addressing all urgent symptoms or issues. Despite these efforts, the LOS is still greater compared to other non-psychiatric hospitalization [15].

Given the influence of the LOS on public health, patient outcomes, and finances, a growing volume of studies have aimed to evaluate factors predicting if an individual has a higher chance for longer hospitalization time. Qualitative synthesis of the available literature was first attempted by Tulloch et al. In their systematic review of 30 studies that analyzed predictors with the LOS of general psychiatric inpatients, several factors were associated with longer LOS including psychosis, female gender, and larger hospital size. On the contrary, voluntary discharge, prospective payment, married status, younger age, and detained status were associated with shorter LOS [5]. Studies were only from the USA, and meta-analysis was not attempted. Another more recent systematic review by Gopalakrishna et al. reported similar findings, with the addition of involuntary admission, mood disorders, use of restraints, and poor treatment response associated with longer LOS [16]. Both studies have contributed towards the anticipation of patients with a higher risk for institutionalization, but do not provide an estimate of the size of the effect on their studied variables on the LOS. While both may be associated with short or long LOS, their comparative magnitude remains uncertain. These are provided by both of the mentioned studies but, to the best of our knowledge, there are no meta-analyses evaluating this adjusted effect of predictors. The importance of the LOS lies not only on the clinical outcome of inpatients, but also on the economic, social, and emotional consequences.

## Research question

In psychiatric inpatients of a mental health institution or hospital, what are the independent predictors of their length of stay?

What is the size of the effect of these independent predictors on the length of stay?

## Objective

The objectives of this systematic review and meta-analysis include:

1. To summarize all factors which influence the length of stay in psychiatric inpatients.
2. To update previous similar studies with more recent published literature.
3. To pool standardized effect sizes to provide an overall point estimate and confidence interval of factors associated with a longer or shorter length of stay.

## Methods

### Protocol registration

The protocol for this systematic review and meta-analysis was registered in PROSPERO (CRD42020172840) on April 28, 2020 (see Additional file 1) and to an independent Research Committee. This study will adhere to the Preferred Reporting Items for Systematic Reviews and Meta-Analyses (PRISMA) guidelines [17] and the checklist with the reported items is provided as an additional file (see Additional file 2).

### Study selection criteria

To be included, studies must fulfill the following criteria:

1. Study design: Prospective or retrospective observational studies.
2. Population characteristics: Inpatients of a psychiatric department/hospital/facility with an Axis I disorder defined by the International Classification of Diseases 10th Edition (ICD-10), or Axis I or II disorder as defined by the Mini International Neuropsychiatric Interview (MINI), Structured Clinical Interview for DSM-5 (SCID), Diagnostic and Statistical Manual of Mental Disorders 4^th^ Edition (DSM-IV), 4th Edition Text-Revised (IV-TR) or 5th Edition (DSM-5).
3. Intervention: Modifiable and non-modifiable factors/determinants/predictors of length of stay (sociodemographic characteristics, medical history, diagnosis, disorder, and treatment-related characteristics).
4. Outcome: Primary outcome is the length of stay in the psychiatric hospital/institution or facility. Only studies which calculated the effect size through reporting of β-coefficients will be included.

There will be no language restrictions for the studies included. Cross-sectional and prospective or retrospective observational studies which do not fulfill all the above criteria for population, intervention, and outcome will be excluded. Duplicate studies assessing the same population which do not report additional data will also be excluded. To reduce the risk of bias by confounders, we will only consider studies reporting the outcomes of interest through adjusted standardized regression coefficients in this meta-analysis.

### Search process

An independent and experienced librarian (NAAV) will carry out a systematic search in PubMed, Ovid MEDLINE, EMBASE, and PsycINFO to find eligible articles between inception and July 2020. The complete search strategy is provided in an additional file (see Additional file 3). The keywords used were generated through Web of Science and Scopus Controlled vocabulary.

Six reviewers working independently and in duplicate will screen all abstracts and select full-text manuscripts for eligibility. Prior to formal abstract screening, a pilot phase between reviewers will be carried out to clarify misunderstandings and ensure comprehension. Chance-adjusted inter-rater agreement for the title/abstract screening and the full-text eligibility will be calculated using Fleiss’ Kappa, considering a value > 0.7 as indicative of good inter-rater reliability [18]. After title and abstract screening, a second phase of full-text eligibility assessment will ensue. Disagreements at this stage will be resolved by consensus, and reasons for exclusion will be documented by the reviewers. If no consensus is reached, decision will be based on the arbitration of a third reviewer. Abstract screening and full-text selection will be completed using DistillerSR [19], a web-based software designed for screening and data extraction.

### Data extraction

A standardized web-based data extraction form will be designed for the extraction of the information of interest from each study. Data collection will be performed independently and in duplicate by the research team. Discrepancy between reviewers regarding the extracted information will be resolved by consensus or intervention of a third reviewer. From each eligible study the following data will be extracted onto the standardized form:

- Study characteristics: Year, country, design, length, type of mental health institution/hospital level, sample size.
- Sociodemographic variables: Age, sex, education, ethnicity, marital status, type of insurance, accommodation, occupation, income status.
- Medical History: Medical or psychiatric disorders in first- or second-degree family members, psychiatric hospitalization history in first- or second-degree family members, previous and number of psychiatric hospitalizations, previous and number of suicide attempts, time from last psychiatric hospitalization, weight, BMI, medical comorbidities.
- Disorder-related: Primary diagnosis, age at diagnosis, time elapsed since diagnosis, severity rated by clinimetry at admission, voluntary or involuntary admission.
- Treatment: Pharmacotherapy, dosage, duration, non-pharmacological therapy.
- Length of stay: Standardized regression coefficients, total variance, confidence interval, standard errors, mean days of length of stay.

Studies which present compounded terms involving more than one of the aforementioned (i.e., diagnosis plus pharmacotherapy, and age plus diagnosis) will not be considered for the data extraction or the statistical analysis. Furthermore, only standardized regression coefficients that have been adjusted for multiple predictors will be obtained from the included studies. If outcomes or data related to predictions were stated to be assessed in the study but were not reported thereafter either in the results or discussion, we will contact the corresponding author twice through e-mail. If the corresponding author does not respond, we will attempt to contact other authors.

### Statistical analysis

We will provide a qualitative synthesis of the included studies involving their design, country, type of mental health hospital or institution, population characteristics, predictors studied, and a summary of the main findings that includes the average, median or interquartile length for the LOS. For the meta-analysis, we will pool the standardized adjusted regression coefficients (*β*), sample sizes, and standard error as a measure of the effect size of predictors relative to length of stay [20, 21]. Studies that only report the unadjusted coefficients will be included for the systematic review but not for the quantitative analysis, in order to reduce bias. If 95% confidence intervals, standard deviations, or the interquartile range are provided instead of the standard error, we will estimate it with the data provided [22]. A priori selected predictors for meta-analysis are presented in Additional file 4. Nonetheless, in order to reduce the possibilities of increased false-positive rates, meta-analysis will only be performed for predictors with at least ten studies reporting the standardized regression coefficients [23]. Details concerning further steps to test for this are presented in the following sections. With the pooled results we will generate point estimates with 95% confidence intervals on the relationship between each individual predictor and the LOS. We will consider a two-tailed value of *p* < 0.05 as statistically significant.

Heterogeneity of the predictors subjected to meta-analysis that have met the described criteria will be assessed through the I^2^ statistic. A value of *I*^2^ > 50% will be taken as indicative of high heterogeneity not explained by chance [24]. Based on the heterogeneity, the results will be presented using a fixed or random-effects model. When low (< 50%), we will perform the statistical analysis using a fixed-effects model. When the heterogeneity is high (> 50%), we will perform the statistical analysis using a random-effects model and conduct thereafter sensitivity analyses to address underlying causes for the significant heterogeneity. This analysis method has been described to reduce the rates of type I error [23]. Specific sensitivity analyses are presented in the next section. All calculations will be performed using the R meta-package [25, 26].

### Quality assessment

Two reviewers (FCN and ESU) of the research team will address the risk of bias in each individual study. Since studies considered for inclusion have an observational nature, we will use the Newcastle-Ottawa Quality Scale (NOS) for evaluating prospective and retrospective cohort studies. Domains that will be evaluated include selection quality (representativeness, ascertainment of exposure), comparability quality, and outcome quality (assessment of the outcome, follow-up). Studies will be rated and deemed of good, fair, or poor quality according to the conversion thresholds from the NOS to the Agency for Health Research and Quality (AHRQ) [27]. Disagreements on risk of bias between reviewers will be resolved by consensus or by the arbitration of a third reviewer (AFGM).

### Publication bias

To assess for publication bias, we will use funnel plots of the effect size compared to the standard error [28]. In the presence of plot asymmetry for a given analysis, we will use the trim-and-fill method to determine the impact of removing smaller studies on the overall estimate and provide adjusted results [29].

### Sensitivity and subgroup analyses

If an appropriate number of studies from diverse countries/continents are found, a subgroup analysis by country, continent, and its income status will be carried out to evaluate if differences observed from the general pooled data are significant in specific countries with lower or higher economic status. We also have pre-planned to perform separate analyses for studies involving only a specific diagnosis (i.e., psychotic disorders and substance abuse) In these cases, meta-analyses of correlation will be performed only for studies of that specific disorder. Other a priori sensitivity analyses will be reported based on the quality of the included studies and meta-bias. For the former, only studies rated as good quality will be included for a subgroup analysis to test for differences with the primary pooled estimate (which includes studies with fair or poor quality). If we obtain point estimates from the quality sensitivity analysis that differ significantly from the overall pooled analysis, we will consider the former with more credibility. Furthermore, if other a priori subgroup or sensitivity analyses report significantly different results in comparison to the overall pooled analysis we will consider the overall quality of the different groups to evaluate the validity of the findings.

### Permutation test

The robustness of the pooled standardized regression coefficients will be tested through permutation tests, which has been a recommended method for recalculation of *p* values and statistical significance [23, 30].

## Discussion

From preliminary searches, we have identified there is a substantial body of evidence that should provide sufficient data in order to generate confident estimates. Our study will only consider for inclusion results reported as adjusted standardized regression coefficients. This will limit our analysis in part as other studies evaluating the outcomes of interest with different statistical methods (i.e., hazard, risk, or odds ratio) will be excluded. The robustness of the estimates could also be negatively influenced by the quality of the included studies, given their observational nature. Although this will be addressed in sensitivity/subgroup analyses explained above, there may be a significantly low number of studies of good quality that would make extrapolation of these conclusions not possible. Finally, the discussion from this study will address the following points involved in the informed-decision process:

- Provide more accurate predictions for mental health institutions, psychiatrists, mental health service providers, patients, and families on the prognosis regarding the length of stay for needed inpatient care.
- Identify patients with a higher probability for prolonged hospitalization in order to plan the necessary interventions for these specific situations.
- Update previous systematic reviews [5, 16].
- Highlight the strengths and limitations from the current available evidence.

## Supplementary Information

**Additional file 1:** PROSPERO.

**Additional file 2:** PRISMA-P 2015 Checklist.

**Additional file 3:** Search Strategy.

**Additional file 4:** Pre-specified Predictors.

## Data Availability

All additional files are available.

## Abbreviations

PRISMA: Preferred Reporting Items for Systematic Review and Meta-Analysis
MINI: Mini International Neuropsychiatric Interview
SCID: Structured Clinical Interview for DSM-5
ICD-10: International Classification of Diseases 10th Edition
DSM-IV: Diagnostic and Statistical Manual of Mental Disorders 4^th^ Edition
DSM-VI-TR: Diagnostic and Statistical Manual of Mental Disorders 4^th^ Edition Text-Revised
DSM-V: Diagnostic and Statistical Manual of Mental Disorders 5^th^ Edition
LOS: Length of stay
NOS: Newcastle-Ottawa Scale
AHRQ: Agency for Health Research and Quality
