# Supplementary material for: Predictors of the length of stay of psychiatric inpatients: protocol for a systematic review and meta-analysis
Source: Syst Rev. 2021 Mar 2;10:65. doi: 10.1186/s13643-021-01616-6 (PMC7927412; doi:10.1186/s13643-021-01616-6)
Supplement: Supplementary file 1 — Additional file 1: PROSPERO. [file 13643_2021_1616_MOESM1_ESM.pdf]

Predictors of Length of Stay of Psychiatric Inpatients: Systematic Review and Meta-Analysis

Erasmo Saucedo, Farid Carranza, Andrea Guerrero, Natalia Tamayo, Neri Álvarez, Juan Millán

To enable PROSPERO to focus on COVID-19 registrations during the 2020 pandemic, this registration record was automatically published exactly as submitted. The PROSPERO team has not checked eligibility.

Citation

Erasmo Saucedo, Farid Carranza, Andrea Guerrero, Natalia Tamayo, Neri Álvarez, Juan Millán. Predictors of Length of Stay of Psychiatric Inpatients: Systematic Review and Meta-Analysis. PROSPERO 2020 CRD42020172840 Available from:
https://www.crd.york.ac.uk/prospero/display_record.php?ID=CRD42020172840

Review question

What are the determinants or predictor factors of length of stay among psychiatric inpatients?

Searches

The following information sources will be addressed in the search strategy: PubMed, MEDLINE, Scopus, Web of Science, EMBASE, Ovid and the Cochrane Central database. An independent and experienced librarian (NA) will perform the search strategy with the collaboration of the main investigators. A systematic search strategy without language restrictions will be performed, which will include Medical Subject Headings (MeSH) terms, as well as specific keywords related to the topic of the research. The search will include any type of scientific article published in a scientific journal between databases from inception and April 2020.

Types of study to be included

Inclusion: Prospective or retrospective cohorts that use a multivariate linear regression to estimate the effects of study variables on length of stay among psychiatric inpatients. Factors associated with length of stay will include sociodemographic characteristics, medical/psychiatric history, disorder-related characteristics and treatment.

Exclusion: Cross-sectional studies, prospective or retrospective cohorts which do not use a multivariate linear analysis to determine the effects of study variables on length of stay among psychiatric inpatients.

Condition or domain being studied

This systematic review will study the impact of modifiable and non-modifiable factors/determinants/predictors on the length of stay among hospitalized patients in psychiatric wards/hospitals. Length of stay is defined as the total duration in days from the day of admission to the day of discharge, which is considerably longer in psychiatric hospitals. Outcomes from this study will be critical to infer and understand causes underlying longer lengths of stay that lead to higher costs, as well as to predict patients who are at risk for extended stays and develop a specialized treatment plan to reduce this.

Participants/population

Inclusion: Patients of a psychiatric ward or hospital, with no age restrictions, and cause of admission is due to psychiatric morbidity.

Exclusion: Outpatients (non-hospitalized) and hospitalized patients in facilities or hospitals not destined to mental health care.

Intervention(s), exposure(s)

Modifiable and non-modifiable factors/determinants/predictors of psychiatric inpatients (sociodemographic characteristics, medical history, diagnosis and disorder-related characteristics).

Comparator(s)/control

Not Relevant

Main outcome(s)

Systematic review: To synthesize the evidence associating exposure variables or risk factors to the length of stay among psychiatric inpatients.

Meta-analysis: Effect size of exposure variables or risk factors on the length of stay among psychiatric inpatients.

* Measures of effect

Risk ratios for prospective studies

Odds ratios for retrospective studies

Additional outcome(s)

None

* Measures of effect

None

Data extraction (selection and coding)

Four reviewers (ES, FC, AG, NT) will work independently and in duplicate to first review all titles and abstracts that were yielded in the search strategy, after this, they will again review full texts of all studies that were considered for inclusion in the previous phase. During the title and abstract reviewing phase, disagreements on inclusion between reviewers will be addressed during the full text review phase, where any kind of disagreement will be resolved by consensus or by the intervention of a third reviewer. Inter-rater agreement will be addressed before each phase.

A standardized web-based data extraction form will be designed for the extraction of the information of interest from each study. Data collection will be performed independently and in duplicate by two members (FC and AG) of the research team, any kind of discrepancy between reviewers regarding the extracted information will be resolved by consensus or intervention of a third reviewer. The following data will be gathered:

- Study characteristics: Year, Country, Design (prospective, retrospective), Mental Health Institution/Hospital Level, Number of patients

- Sociodemographic characteristics: Age, ethnicity, education level, employment, nationality, relationship status, work area, accomodation, economic status, type of insurance,

- Medical history: Previous psychiatric hospitalizations, recent hospitalizations, suicide attempts, medical/ psychiatric comorbidities, weight, body-mass index.

- Disorder-related characteristics: Primary psychiatric diagnosis, age at diagnosis, time elapsed since diagnosis, severity rated by clinimetry at admission, type of admission (voluntary vs involuntary; planned vs urgent)

- Length of stay from multivariate linear regression: regression coefficients, total variance, confidence interval, standard errors, mean days of length of stay

Risk of bias (quality) assessment

Two members of the research team (ES and FC) will address the risk of bias in each individual study both independently and in duplicate. Since studies considered for inclusion have an observational nature, we will use the Newcastle-Ottawa Quality Scale (NOS) for evaluating prospective and retrospective cohort studies. Domains that will be evaluated include: Selection Quality (representativeness, ascertainment of exposure), Comparability Quality and Outcome Quality (assessment of the outcome, follow-up). Studies will be rated and deemed of Good, Fair or Poor Quality according to the conversion thresholds from the NOS to the

Agency for Health Research and Quality (AHRQ). Disagreements on risk of bias between reviewers will be resolved by consensus or by the arbitration of a third reviewer (AG).

Strategy for data synthesis

A narrative synthesis of the included studies will be provided in a table describing the year, country, design (prospective or retrospective), sample size, demographic characteristics, mean length of stay (if available) and main diagnoses. Meta-analysis will be performed using regression coefficients from multivariate analyses. The pooled estimate of the effect will involve the inverse variance of regression (beta) coefficients will involve as weight, calculated through 95% confidence intervals, p-value, standard error or sample size. Comparisons will be carried out when regression coefficients of a given variable are reported in reference to the same comparator in multiple studies (i.e., female compared to male). Heterogeneity will be assessed through the I^2 and $?^2$ statistics, considering a cutoff value of 50% and 0.05 as statistically significant heterogeneity not explained by chance. Fixed-effect models will be used in cases of low to absent heterogeneity.

Analysis of subgroups or subsets

If an appropriate number of studies from diverse countries/continents are found, a subgroup analyses by country, continent and its income status will be carried out to evaluate if differences observed from the general pooled data are significant in specific countries with lower or higher economic status. It is pre-planned to perform separate analyses for studies involving only a specific diagnosis (i.e., psychotic disorders). In these cases, meta-analyses of correlation coefficients will be performed between the previously mentioned variables and the length of stay, only for studies of that specific disorder.

Contact details for further information

Farid Carranza Navarro
farid.carranza@gmail.com

Organisational affiliation of the review

Centro de Neurociencias Avanzadas UANL (CENAU)
Department of Psychiatry
University Hospital "Dr. José E. González"
Universidad Autónoma de Nuevo León (UANL)
http://www.medicina.uanl.mx/psiquiatria/

Review team members and their organisational affiliations

Dr Erasmo Saucedo. Centro de Neurociencias Avanzadas UANL (CENAU), Department of Psychiatry, University Hospital
Dr Farid Carranza. Centro de Neurociencias Avanzadas UANL (CENAU), Department of Psychiatry, University Hospital
Dr Andrea Guerrero. Centro de Neurociencias Avanzadas UANL (CENAU), Department of Psychiatry, University Hospital
Miss Natalia Tamayo. Centro de Neurociencias Avanzadas UANL (CENAU), Department of Psychiatry, University Hospital
Dr Neri Álvarez. Plataforma INVEST Medicina UANL-Ker Unit Mayo Clinic (KER Unit Mexico), Research Subdirectorate, Faculty of Medicine, Universidad Autónoma de Nuevo León
Mr Juan Millán. Plataforma INVEST Medicina UANL-Ker Unit Mayo Clinic (KER Unit Mexico), Research Subdirectorate, Faculty of Medicine, Universidad Autónoma de Nuevo León

Collaborators

Dr René Rodríguez. Plataforma INVEST Medicina UANL-Ker Unit Mayo Clinic (KER Unit Mexico), Research Subdirectorate, Faculty of Medicine, Universidad Autónoma de Nuevo León

Type and method of review

Epidemiologic, Meta-analysis, Prognostic, Systematic review

Anticipated or actual start date

01 April 2020

Anticipated completion date

01 September 2020

Funding sources/sponsors

None

Grant number(s)

None

Conflicts of interest

None known

Language

English

Country

Mexico

Stage of review

Review Ongoing

Subject index terms status

Subject indexing assigned by CRD

Subject index terms

MeSH headings have not been applied to this record

Date of registration in PROSPERO

28 April 2020

Date of first submission

18 March 2020

Stage of review at time of this submission

The review has not started

**Stage**	**Started**	**Completed**
Preliminary searches	No	No
Piloting of the study selection process	No	No
Formal screening of search results against eligibility criteria	No	No
Data extraction	No	No
Risk of bias (quality) assessment	No	No
Data analysis	No	No

The record owner confirms that the information they have supplied for this submission is accurate and complete and they understand that deliberate provision of inaccurate information or omission of data may be construed as scientific misconduct.

The record owner confirms that they will update the status of the review when it is completed and will add publication details in due course.

Versions

28 April 2020

PROSPERO
This information has been provided by the named contact for this review. CRD has accepted this information in good faith and registered the review in PROSPERO. The registrant confirms that the information supplied for this submission is accurate and complete. CRD bears no responsibility or liability for the content of this registration record, any associated files or external websites.
