# Supplementary material for: Predictors of the length of stay of psychiatric inpatients: protocol for a systematic review and meta-analysis
Source: Syst Rev. 2021 Mar 2;10:65. doi: 10.1186/s13643-021-01616-6 (PMC7927412; doi:10.1186/s13643-021-01616-6)
Supplement: Supplementary file 3 — Additional file 3: Search Strategy. [file 13643_2021_1616_MOESM3_ESM.docx]

**Search Strategy**

A comprehensive search will be conducted to find eligible articles in several databases from inception to July 2020. These databases will include PubMed, MEDLINE, Scopus, Google Scholar, EMBASE and PsycINFO. A systematic search strategy without language restrictions will be performed, which will include Medical Subject Headings (MeSH) terms, as well as specific keywords related to the topic of the research. The search will include any type of scientific article published in a scientific journal between each databases inception date and July 2020.

The search will be made with the following terms:

**Population**: Hospital, Mental OR Hospital, Psychiatric OR Hospitals, Mental OR Institution, Mental OR Institutions, Mental OR Mental Hospital OR Mental Hospitals OR Mental Institution OR Mental Institutions OR Psychiatric Hospital OR Psychiatric Hospitals

**Intervention**: Predictors OR Risk Factors OR Risk Factor OR Factors, Risk OR Factor, Risk OR Population at Risk OR Populations at Risk OR Risk, Population at OR Risk, Populations at OR Determinant, Epidemiologic OR Determinants, Epidemiologic OR Epidemiologic Determinants OR Epidemiologic Determinant OR Epidemiologic Factor OR Factor, Epidemiologic OR Epidemiologic Factors

**Outcomes**: Length of Stay OR Hospital Stay OR Hospital Stays OR Stay Length OR Stay, Hospital OR Stays Hospitals

**Not to Include**: Outpatients OR Outpatient OR Emergency Psychiatric Service OR Emergency Psychiatric Services OR Emergency Service, Psychiatric OR Psychiatric Emergency Service OR Psychiatric Emergency Services OR Psychiatric Service, Emergency OR Psychiatric Services, Emergency OR Service, Emergency Psychiatric OR Service, Psychiatric Emergency OR Services, Emergency Psychiatric OR Services, Psychiatric Emergency
