# Supplementary material for: Predictors of the length of stay of psychiatric inpatients: protocol for a systematic review and meta-analysis
Source: Syst Rev. 2021 Mar 2;10:65. doi: 10.1186/s13643-021-01616-6 (PMC7927412; doi:10.1186/s13643-021-01616-6)
Supplement: Supplementary file 4 — Additional file 4: Pre-specified Predictors. [file 13643_2021_1616_MOESM4_ESM.docx]

**Additional File 4**

The following predictors will be *a priori* considered during the statistical analysis to be subjected to meta-analysis, under the conditions specified in the protocol. Furthermore, pooled results from this analysis will also be subject to heterogeneity assessment and permutation tests.

1. Sex
2. Education
3. Ethnicity/Race
4. Marital status
5. Insurance
6. Economic status
7. Occupation
8. Schizophrenia spectrum and psychotic disorders
9. Mood disorders
10. Substance abuse disorders
11. Stress related disorders
12. Personality disorders
13. Prior hospitalizations
14. Medical comorbidities
15. Antipsychotics
16. Antidepressants
17. Sedatives
18. Mood stabilizers
